# Supplementary material for: Loss of TMEM106B and PGRN leads to severe lysosomal abnormalities and neurodegeneration in mice
Source: EMBO Rep. 2020 Aug 10;21(10):e50219. doi: 10.15252/embr.202050219 (PMC7534636; doi:10.15252/embr.202050219)
Supplement: Supplementary file 1 — Expanded View Figures PDF [file EMBR-21-e50219-s001.pdf]

Expanded View Figures

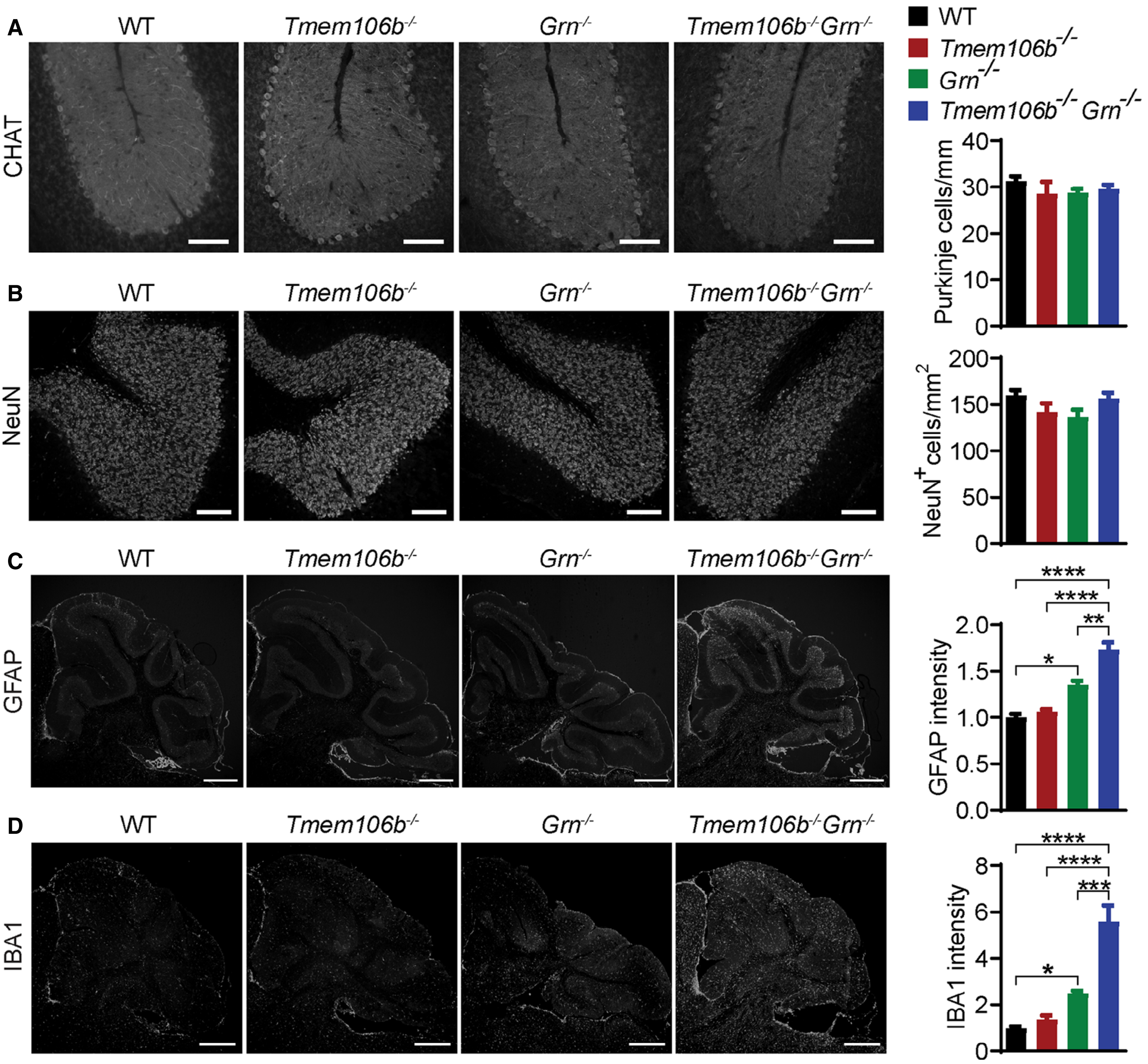

Figure EV1. Increased gliosis in the cerebellum of *Tmem106b*$^{-/-}$*Grn*$^{-/-}$ mice.

A–D Immunostaining of CHAT, NeuN, GFAP, and IBA1 in cerebellum sections from 5-month-old WT, *Tmem106b*$^{-/-}$, *Grn*$^{-/-}$, and *Tmem106b*$^{-/-}$*Grn*$^{-/-}$ mice. The number of Purkinje cells/length of linear line of Purkinje cell arrangement (A), the number of granule cells in the stained sections (B) and GFAP (C) and IBA1 (D) intensities were quantified. $n = 3$. Data presented as mean ± SEM. One-way ANOVA tests with Bonferroni's multiple comparisons: *$P < 0.05$, **$P < 0.01$, ***$P < 0.001$, ****$P < 0.0001$. (A, B) Scale bar = 100 μm. (C, D) Scale bar = 500 μm.

© 2020 The Authors

Figure EV2. Gene Ontology analysis of RNA-Seq data from spinal cord of *Tmem106b*$^{-/-}$*Grn*$^{-/-}$ mouse.

A Gene enrichment analysis using cellular component, KEGG pathways, molecular function, and biological process for upregulated differentially expressed genes (DEGs) in the *Tmem106b*$^{-/-}$*Grn*$^{-/-}$ (DKO) spinal cord samples compared to WT with FDR ≤ 10%.

B Gene enrichment analysis using cellular component, molecular function, and biological process for downregulated differentially expressed genes (DEGs) in the *Tmem106b*$^{-/-}$*Grn*$^{-/-}$ (DKO) spinal cord samples compared to WT with FDR ≤ 10%.

C Venn diagrams showing the overlap among differentially expressed genes (DEGs) (WT vs *Tmem106b*$^{-/-}$*Grn*$^{-/-}$) identified by RNA-Seq, known DAM genes, and mouse lysosomal genes. The odd ratios between DEGs vs non-DEGs for DAM genes and lysosome genes are 78.8 (P-value < 2.2e−16) and 9.0 (P-value < 2.2e−16), respectively. Fisher's exact test.

D Venn diagrams showing the overlap among up- or downregulated differentially expressed genes (DEGs) in the *Tmem106b*$^{-/-}$*Grn*$^{-/-}$ spinal cord samples identified by RNA-Seq, known DAM genes, and lysosomal genes.

 © 2020 The Authors

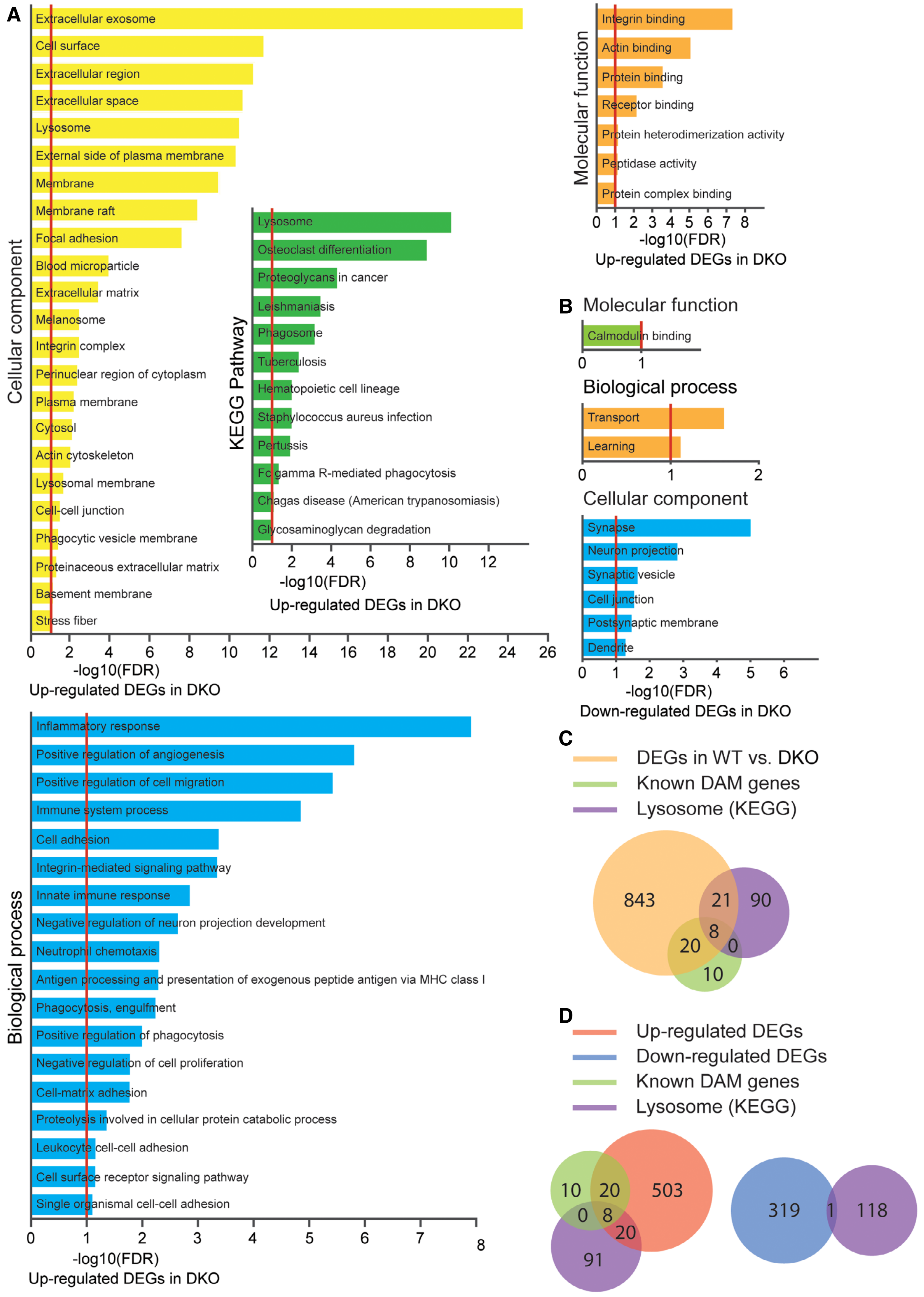
A
Cellular component
Extracellular exosome
Cell surface
Extracellular region
Extracellular space
Lysosome
External side of plasma membrane
Membrane
Membrane raft
Focal adhesion
Blood microparticle
Extracellular matrix
Melanosome
Integrin complex
Perinuclear region of cytoplasm
Plasma membrane
Cytosol
Actin cytoskeleton
Lysosomal membrane
Cell-cell junction
Phagocytic vesicle membrane
Proteinaceous extracellular matrix
Basement membrane
Stress fiber
-log10(FDR)
Up-regulated DEGs in DKO
KEGG Pathway
Lysosome
Osteoclast differentiation
Proteoglycans in cancer
Leishmaniasis
Phagosome
Tuberculosis
Hematopoietic cell lineage
Staphylococcus aureus infection
Pertussis
Fc gamma R-mediated phagocytosis
Chagas disease (American trypanosomiasis)
Glycosaminoglycan degradation
-log10(FDR)
Up-regulated DEGs in DKO
Molecular function
Integrin binding
Actin binding
Protein binding
Receptor binding
Protein heterodimerization activity
Peptidase activity
Protein complex binding
-log10(FDR)
Up-regulated DEGs in DKO
B
Molecular function
Calmodulin binding
Biological process
Transport
Learning
Cellular component
Synapse
Neuron projection
Synaptic vesicle
Cell junction
Postsynaptic membrane
Dendrite
-log10(FDR)
Down-regulated DEGs in DKO
Biological process
Inflammatory response
Positive regulation of angiogenesis
Positive regulation of cell migration
Immune system process
Cell adhesion
Integrin-mediated signaling pathway
Innate immune response
Negative regulation of neuron projection development
Neutrophil chemotaxis
Antigen processing and presentation of exogenous peptide antigen via MHC class I
Phagocytosis, engulfment
Positive regulation of phagocytosis
Negative regulation of cell proliferation
Cell-matrix adhesion
Proteolysis involved in cellular protein catabolic process
Leukocyte cell-cell adhesion
Cell surface receptor signaling pathway
Single organismal cell-cell adhesion
-log10(FDR)
Up-regulated DEGs in DKO
C
DEGs in WT vs. DKO
Known DAM genes
Lysosome (KEGG)
843
21
90
8
20
0
10
D
Up-regulated DEGs
Down-regulated DEGs
Known DAM genes
Lysosome (KEGG)
10
20
503
0
8
20
91
319
1
118

Figure EV2.

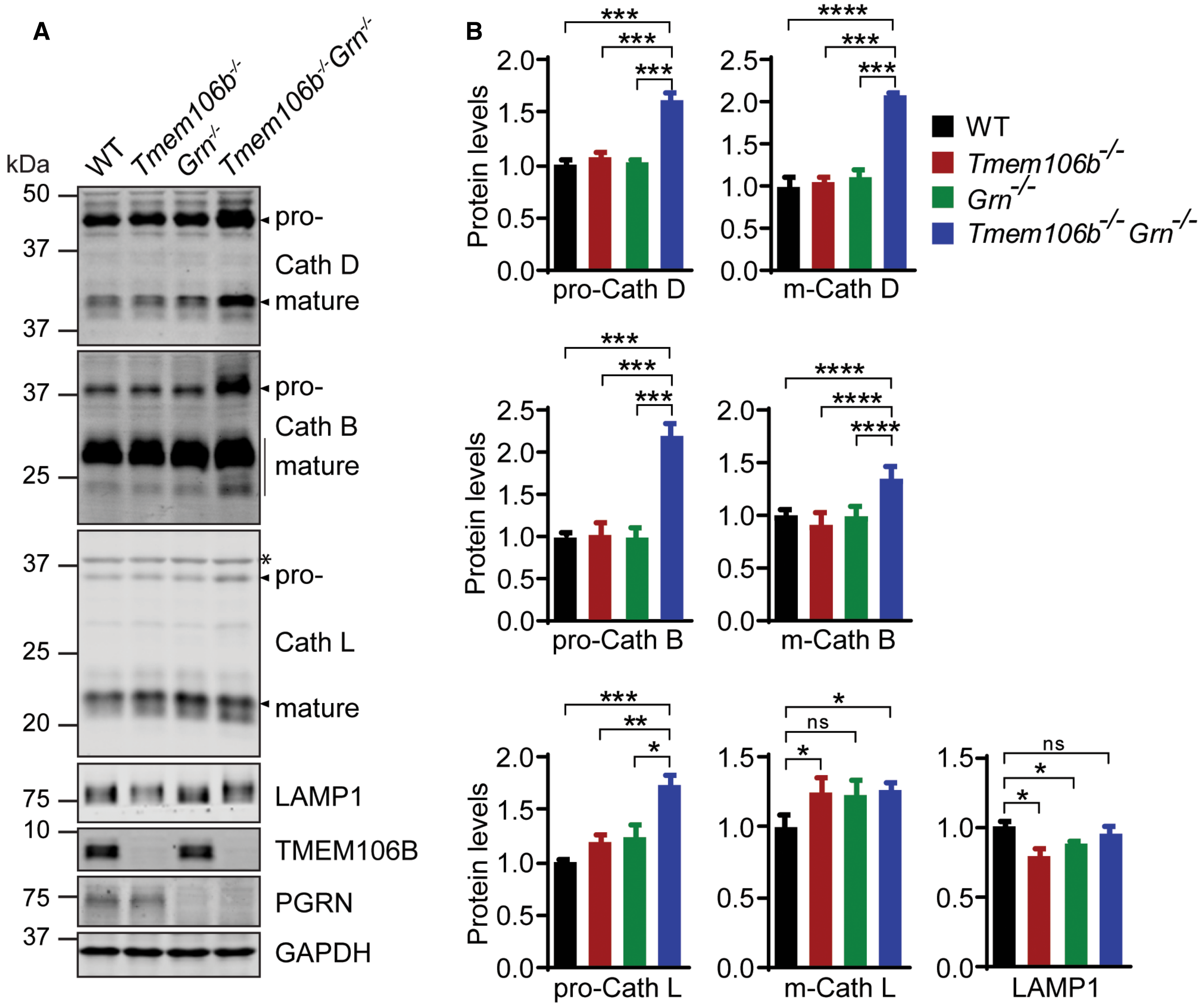

Figure EV3. Western blot analysis of lysosomal protein levels in *Tmem106b*$^{-/-}$*Grn*$^{-/-}$ mouse brain lysate.

A, B Western blot analysis of lysosomal proteins in brain lysates of 5-month-old WT, *Tmem106b*$^{-/-}$, *Grn*$^{-/-}$, and *Tmem106b*$^{-/-}$*Grn*$^{-/-}$ mice. Asterisk indicates non-specific bands. $n = 3$. Data presented as mean ± SEM. One-way ANOVA tests with Bonferroni's multiple comparisons: *$P < 0.05$, **$P < 0.01$, ***$P < 0.001$, ****$P < 0.0001$, ns, no significance.

 © 2020 The Authors

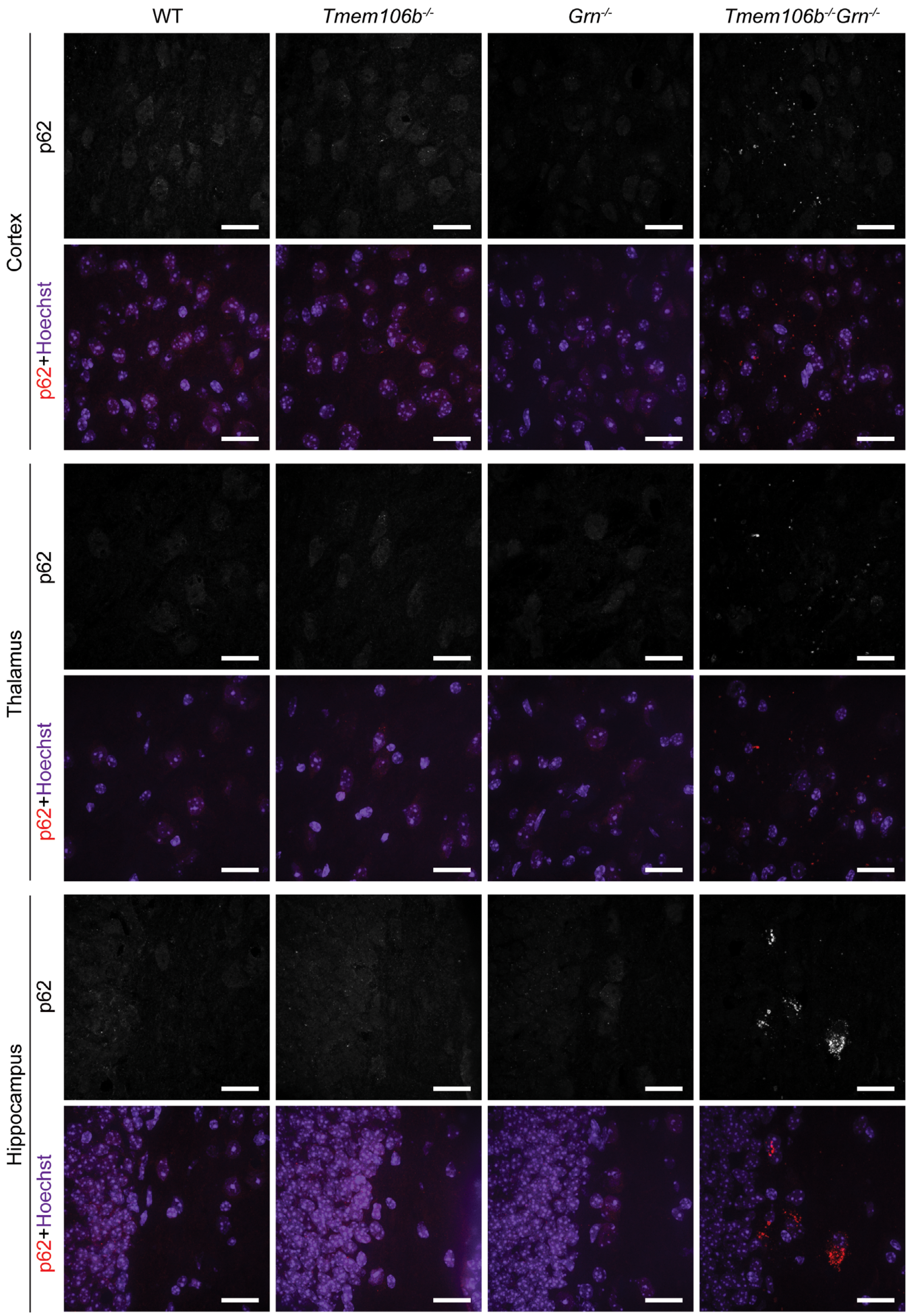

Figure EV4. Accumulation of p62 aggregates in the brain of *Tmem106b*$^{-/-}$*Grn*$^{-/-}$ mice.

Immunostaining of p62 in brain sections from 5-month-old WT, *Tmem106b*$^{-/-}$, *Grn*$^{-/-}$, and *Tmem106b*$^{-/-}$*Grn*$^{-/-}$ mice, and images were taken from cortex, thalamus, and hippocampus regions. $n = 3$. Scale bar = 20 µm.

Source data are available online for this figure.

© 2020 The Authors

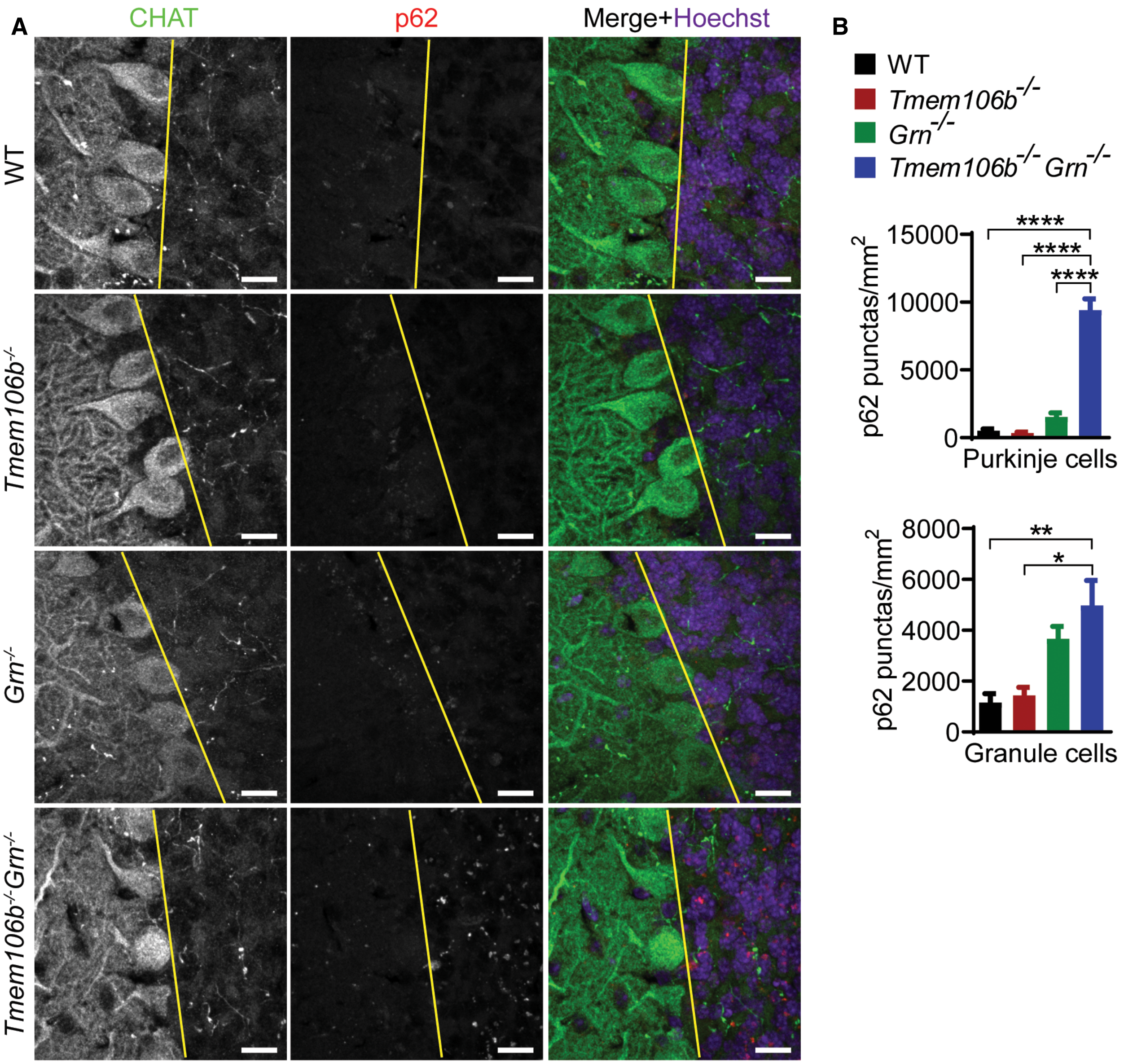

Figure EV5. Accumulation of p62 aggregates in the cerebellum of *Tmem106b*$^{-/-}$*Grn*$^{-/-}$ mice.

A, B Immunostaining of p62 and CHAT in cerebellum sections from 5-month-old WT, *Tmem106b*$^{-/-}$, *Grn*$^{-/-}$, and *Tmem106b*$^{-/-}$*Grn*$^{-/-}$ mice. Yellow line indicates the border between Purkinje cell layer and granule cell layer. Scale bar = 15 μm. (B) Number of p62 puncta in Purkinje cell layer and granule cell layer was quantified. $n = 3$. Data presented as mean ± SEM. One-way ANOVA tests with Bonferroni's multiple comparisons: *$P < 0.05$, **$P < 0.01$, ****$P < 0.0001$.

© 2020 The Authors
