# Supplementary material for: Loss of TMEM106B and PGRN leads to severe lysosomal abnormalities and neurodegeneration in mice
Source: EMBO Rep. 2020 Aug 10;21(10):e50219. doi: 10.15252/embr.202050219 (PMC7534636; doi:10.15252/embr.202050219)
Supplement: Supplementary file 3 — Table EV2 [file EMBR-21-e50219-s003.pdf]

Table EV2: List of lysosome genes and DAM genes for the RNA-seq analysis

KEGG Lysosome genes mmu04142						DAM genes	
**Gene List**				**Upregulated**	**Downregulated**	**Gene list**	**Upregulated**
Abca2	Cd63	Glb1	Npc2	Atp6v0d2	Ap1s2	Ank	Apoe
Abcb9	Cd68	Gm2a	Pla2g15	Cd63		Apoe	Axl
Acp2	Cln3	Gnptab	Ppt1	Cd68		Axl	B2m
Acp5	Cln5	Gnptg	Ppt2	Cln5		B2m	Ccl6
Aga	Clta	Gns	Psap	Ctsa		Cadm1	Cd52
Ap1b1	Cltb	Gusb	Psapl1	Ctsb		Ccl2	Cd63
Ap1g1	Cltc	Hexa	Scarb2	Ctsc		Ccl6	Cd68
Ap1g2	Ctns	Hexb	Sgsh	Ctsd		Cd52	Cd9
Ap1m1	Ctsa	Hgsnat	Slc11a1	Ctsh		Cd63	Clec7a
Ap1m2	Ctsb	Hyal1	Slc11a2	Ctsl		Cd68	Csf1
Ap1s1	Ctsc	Hyal2	Slc17a5	Ctss		Cd9	Cst7
Ap1s2	Ctsd	Hyal3	Smpd1	Ctsz		Clec7a	Cstb
Ap1s3	Ctse	Hyal4	Sort1	Dnase2a		Csf1	Ctsa
Ap3b1	Ctsf	Hyal5	Spam1	Glb1		Cst7	Ctsb
Ap3b2	Ctsg	Hyal6	Sumf1	Gns		Cstb	Ctsd
Ap3d1	Ctsh	Ids	Tcirg1	Gusb		Ctsa	Ctsl
Ap3m1	Ctsj	Idua	Tpp1	Hexa		Ctsb	Ctsz
Ap3m2	Ctsk	Igf2r		Hexb		Ctsd	Cx3cr1
Ap3s1	Ctsl	Lamp1		Lamp2		Ctsl	Gusb
Ap3s2	Ctsm	Lamp2		Laptm5		Ctsz	H2-D1
Ap4b1	Ctso	Lamp3		Lgmn		Cx3cr1	Hif1a
Ap4e1	Ctss	Laptm4a		Lipa		Gusb	Itgax
Ap4m1	Ctsw	Laptm4b		Man2b1		H2-D1	Lpl
Ap4s1	Ctsz	Laptm5		Naglu		Hif1a	Lyz2
Arsa	Dnase2a	Lgmn		Npc2		Itgax	Spp1
Arsb	Dnase2b	Lipa		Sumf1		Lilrb4	Tgfbr1
Arsg	Entpd4	Litaf		Tcirg1		Lpl	Trem2
Asah1	Entpd4b	M6pr		Tpp1		Lyz2	Tyrobp
Atp6ap1	Fuca1	Man2b1				P2ry12	
Atp6v0a1	Fuca2	Manba				Serinc3	
Atp6v0a2	Gaa	Mcoln1				Serpine2	
Atp6v0a4	Galc	Mfsd8				Spp1	
Atp6v0b	Galns	Naga				Tgfbr1	
Atp6v0c	Gba	Naglu				Timp2	
Atp6v0d1	Gga1	Nagpa				Tmem119	
Atp6v0d2	Gga2	Napsa				Trem2	
Atp6v1h	Gga3	Neu1				Txnip	
Cd164	Gla	Npc1				Tyrobp	

Source of DAM genes:

1. Keren-Shaul H, , *et al.* (2017) *Cell* **169:** 1276-1290 e1217

2. Deczkowska A,et al (2018) *Cell* **173:** 1073-1081

3. Krasemann S, *et al.* (2017) *Immunity* **47:** 566-581 e569
