# Supplementary material for: Loss of TMEM106B and PGRN leads to severe lysosomal abnormalities and neurodegeneration in mice
Source: EMBO Rep. 2020 Aug 10;21(10):e50219. doi: 10.15252/embr.202050219 (PMC7534636; doi:10.15252/embr.202050219)
Supplement: Supplementary file 5 — Movie EV1 [file EMBR-21-e50219-s005.zip › Movie EV1 Legend.docx]

**Movie EV1.** An example of 5-month-old *Tmem106b^-/-^Grn^-/-^*(DKO) mice showing reduced motor activity compared to WT littermate control.
