# Supplementary material for: Loss of TMEM106B and PGRN leads to severe lysosomal abnormalities and neurodegeneration in mice
Source: EMBO Rep. 2020 Aug 10;21(10):e50219. doi: 10.15252/embr.202050219 (PMC7534636; doi:10.15252/embr.202050219)
Supplement: Supplementary file 6 — Movie EV2 [file EMBR-21-e50219-s006.zip › Movie EV2 Legend.docx]

**Movie EV2.** An example of 5-month-old *Tmem106b^-/-^Grn^-/-^*(DKO) mice with hind limb paralysis compared to WT littermate control.
