# Supplementary figures and images for: Loss of TMEM106B and PGRN leads to severe lysosomal abnormalities and neurodegeneration in mice
Source: EMBO Rep. 2020 Aug 10;21(10):e50219. doi: 10.15252/embr.202050219 (PMC7534636; doi:10.15252/embr.202050219)

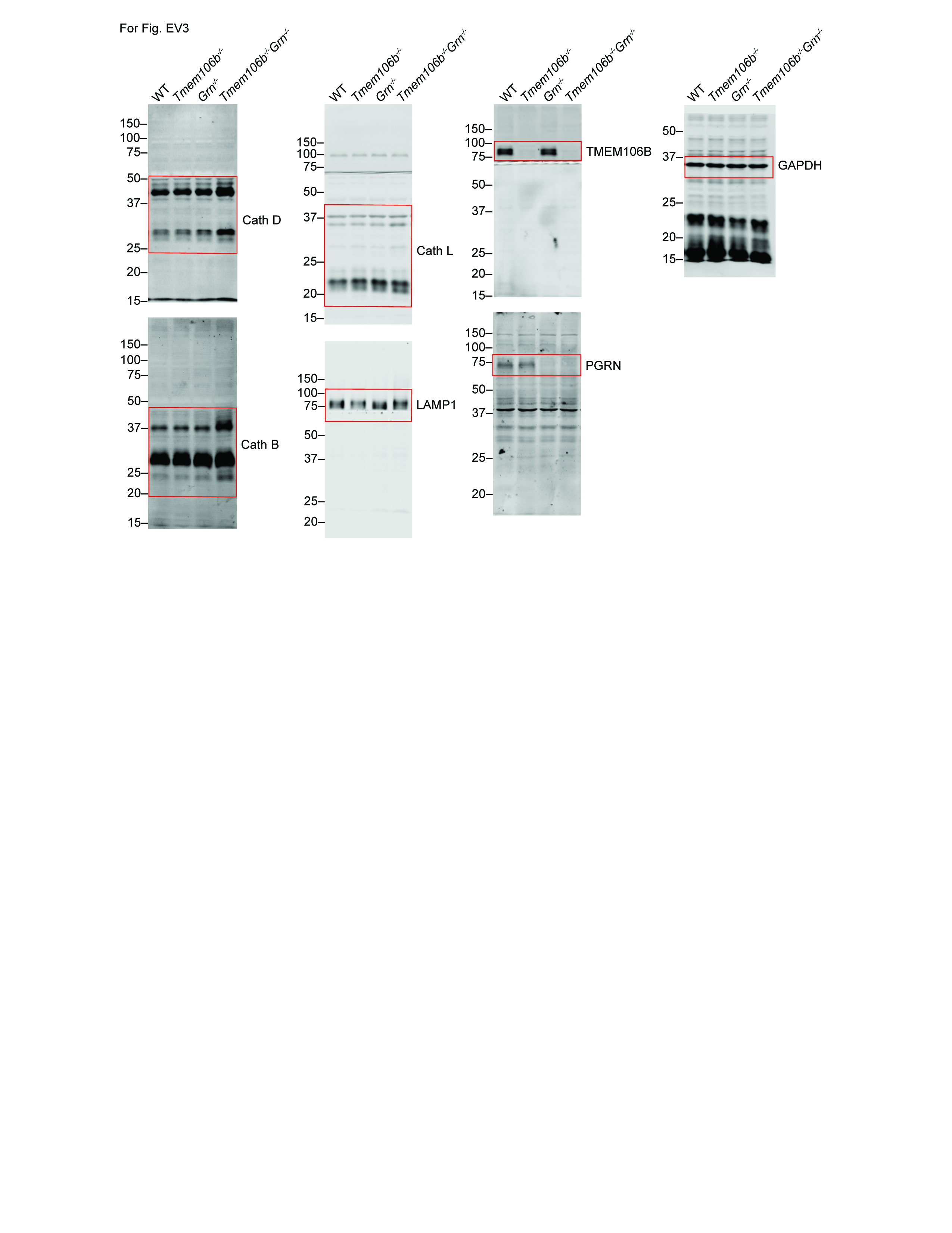

Supplement: Supplementary file 7 — Source Data for Expanded View [file EMBR-21-e50219-s013.tif]

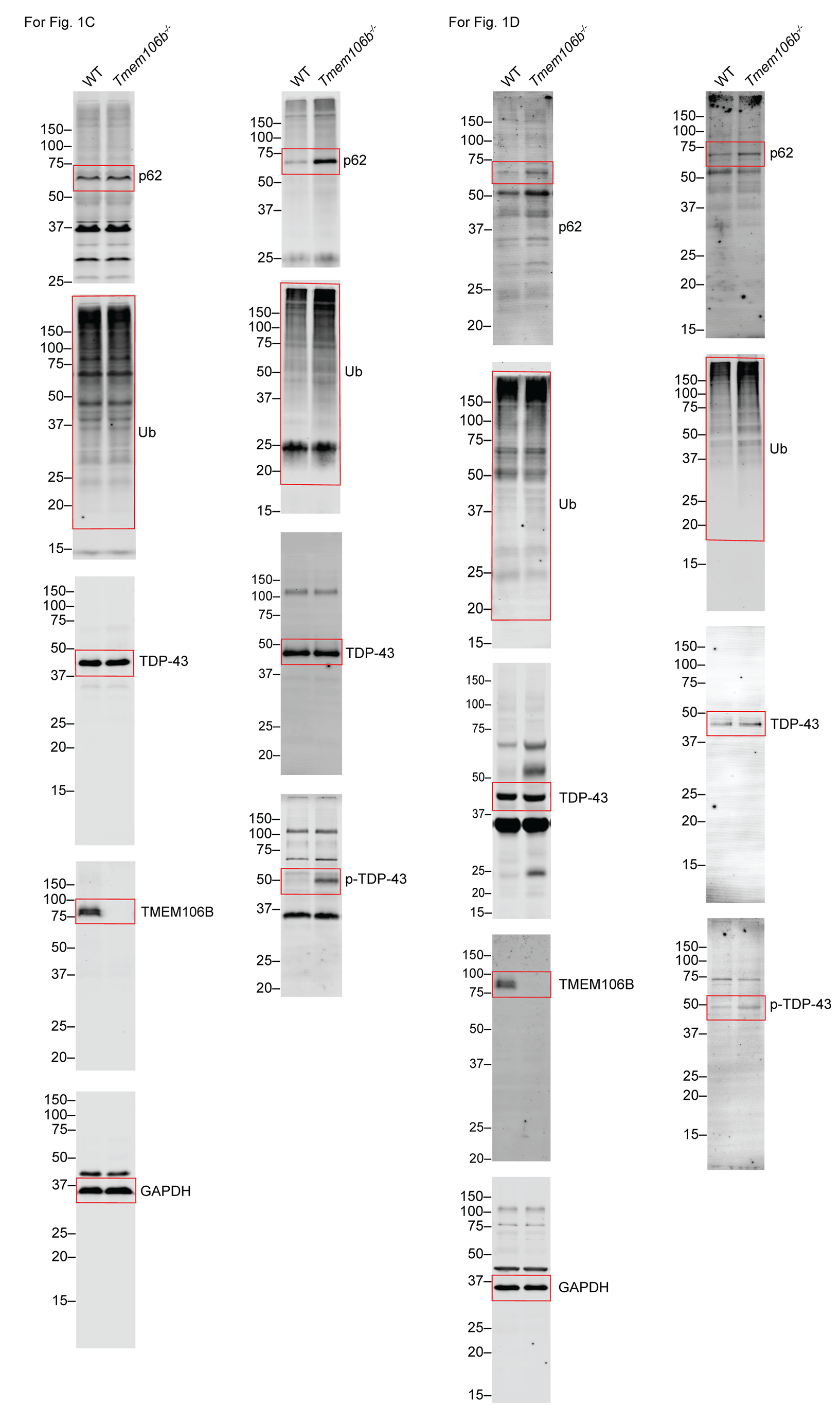

Supplement: Supplementary file 9 — Source Data for Figure 1 [file EMBR-21-e50219-s007.tif]

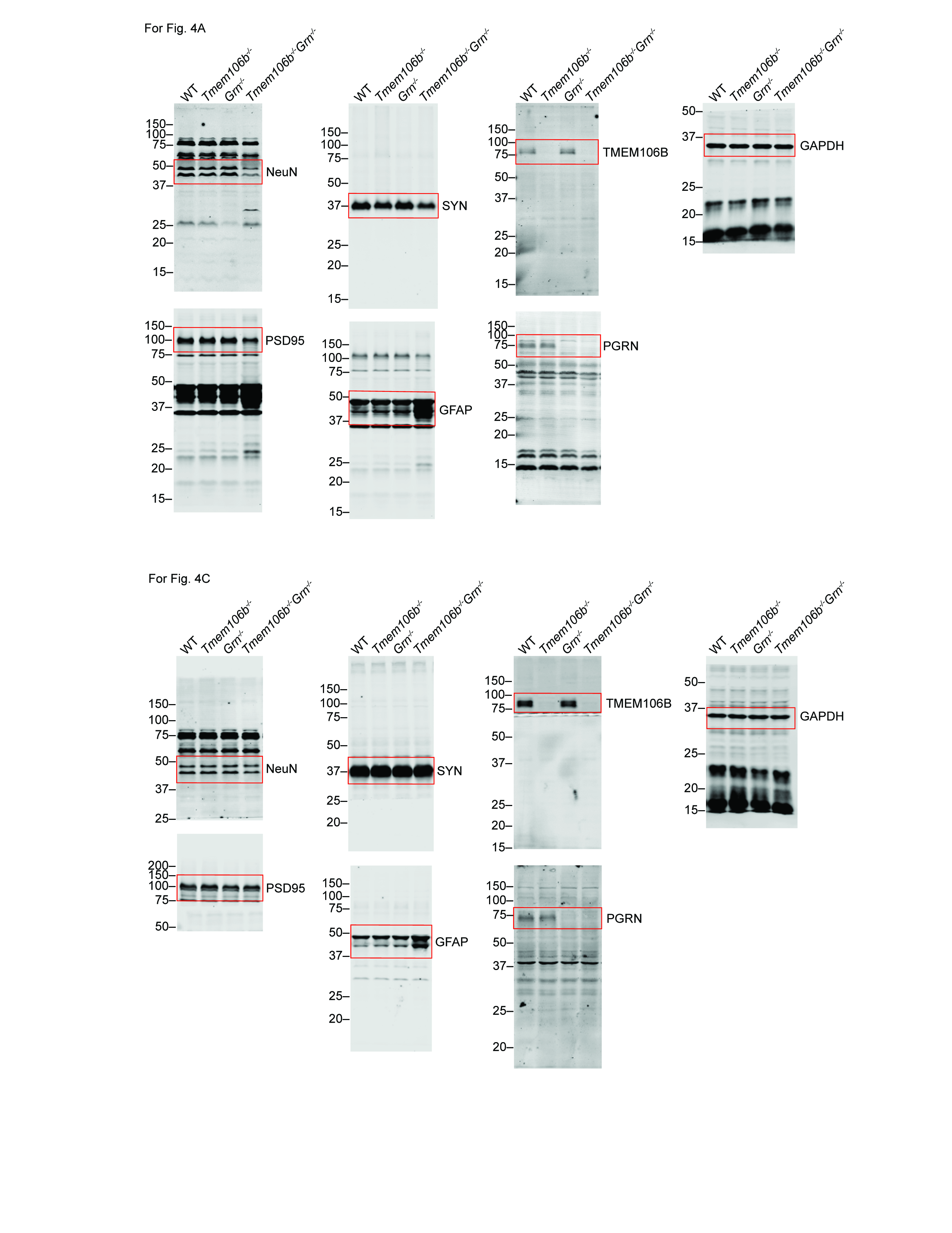

Supplement: Supplementary file 10 — Source Data for Figure 4 [file EMBR-21-e50219-s008.tif]

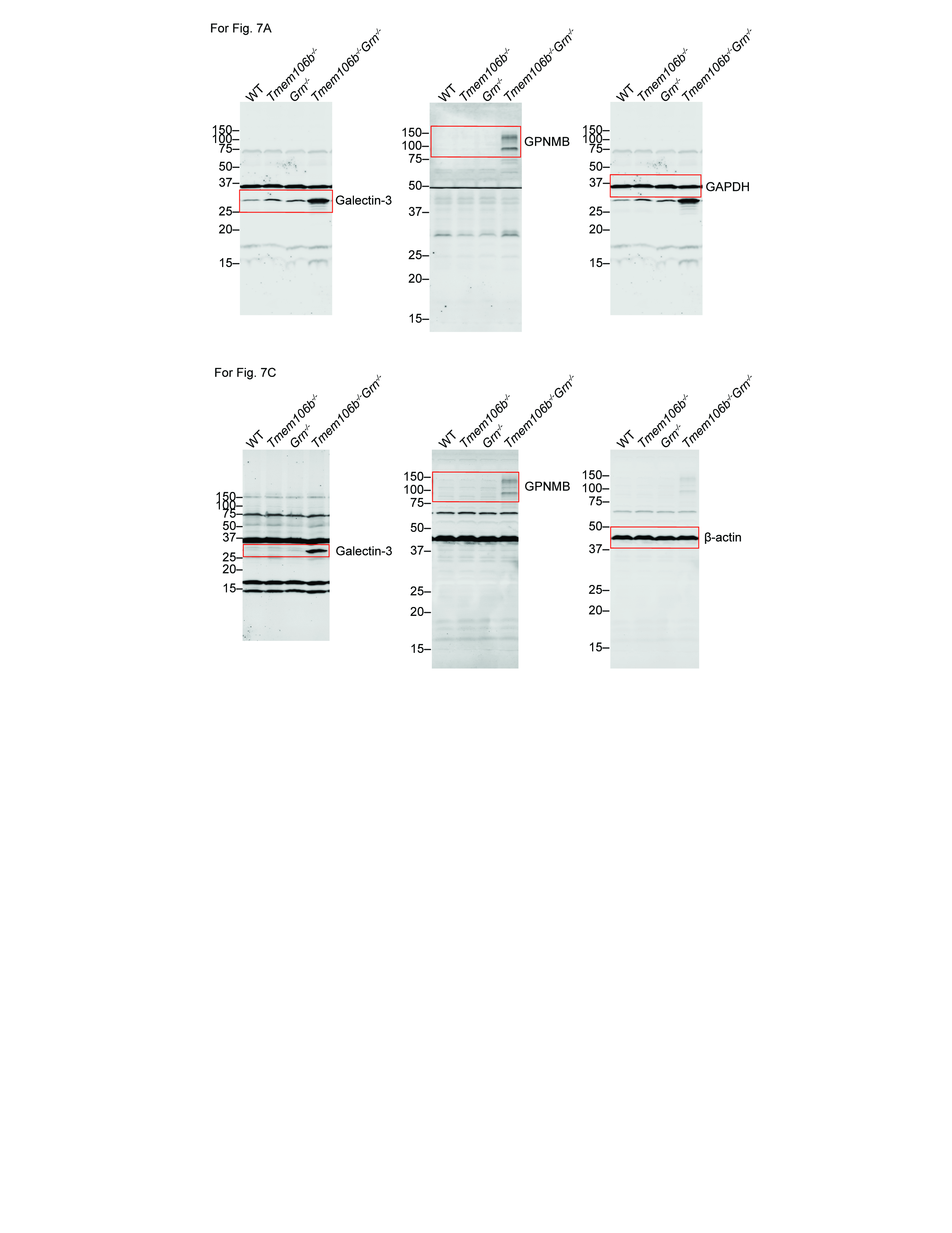

Supplement: Supplementary file 11 — Source Data for Figure 7 [file EMBR-21-e50219-s009.tif]

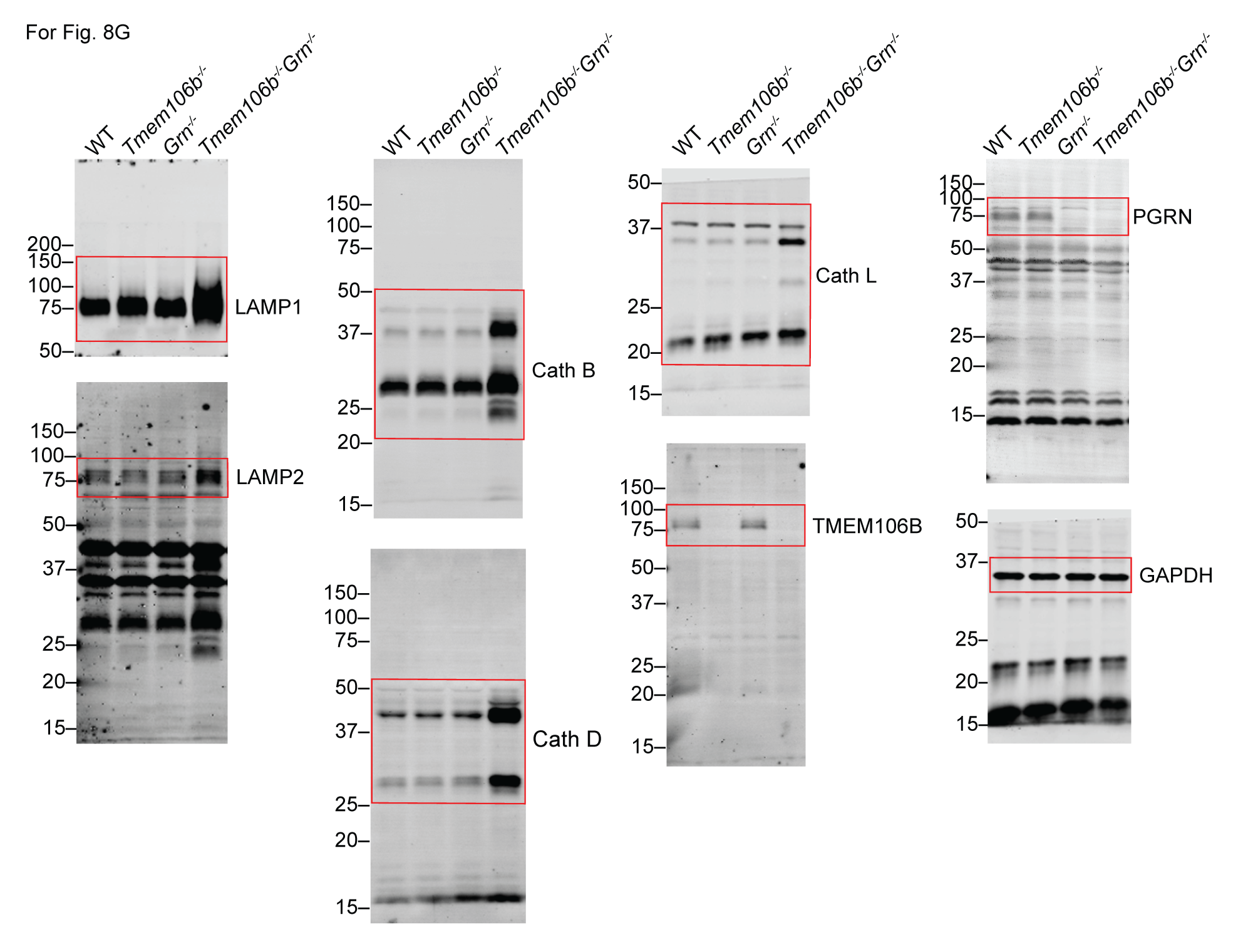

Supplement: Supplementary file 12 — Source Data for Figure 8 [file EMBR-21-e50219-s010.tif]

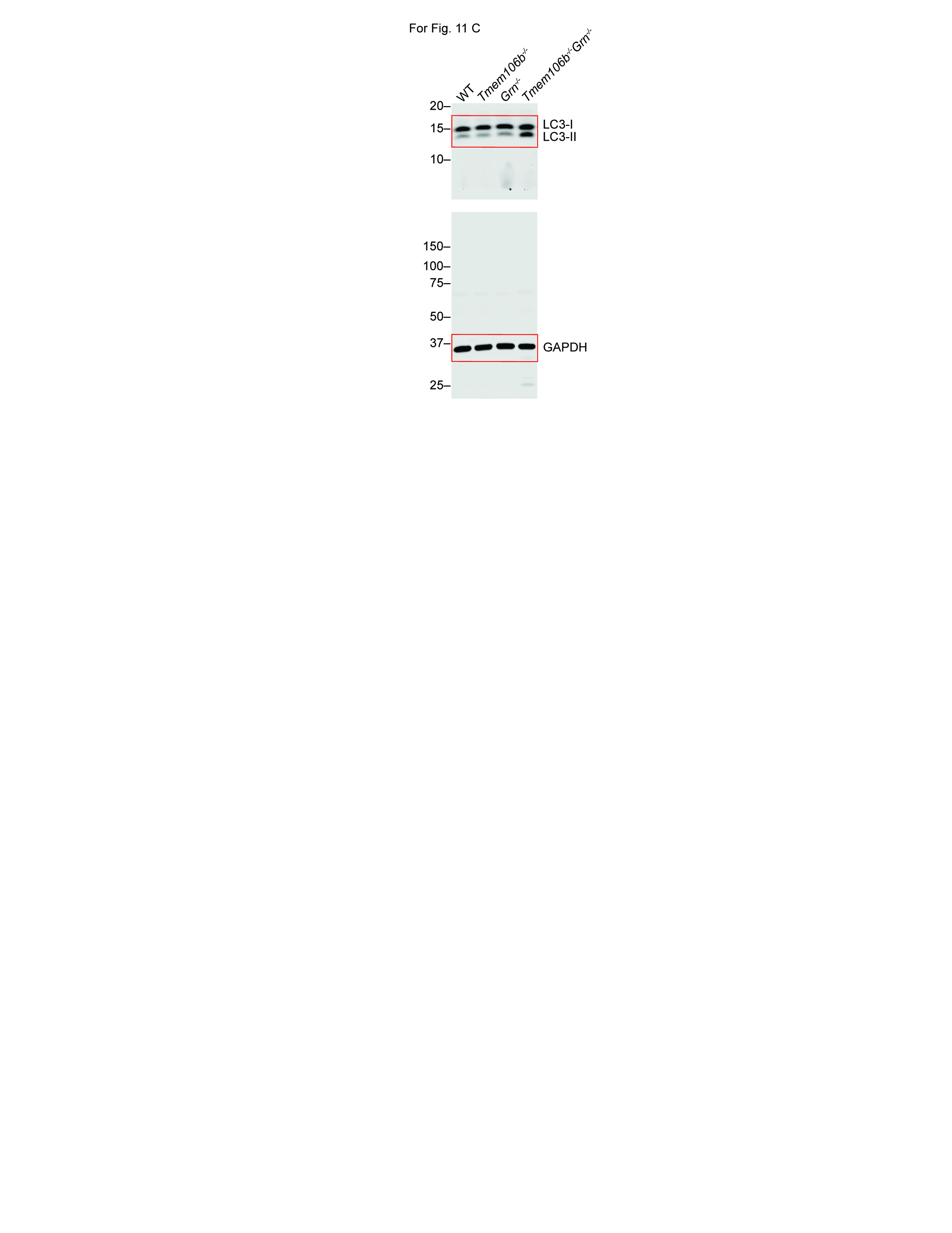

Supplement: Supplementary file 13 — Source Data for Figure 11 [file EMBR-21-e50219-s011.tif]

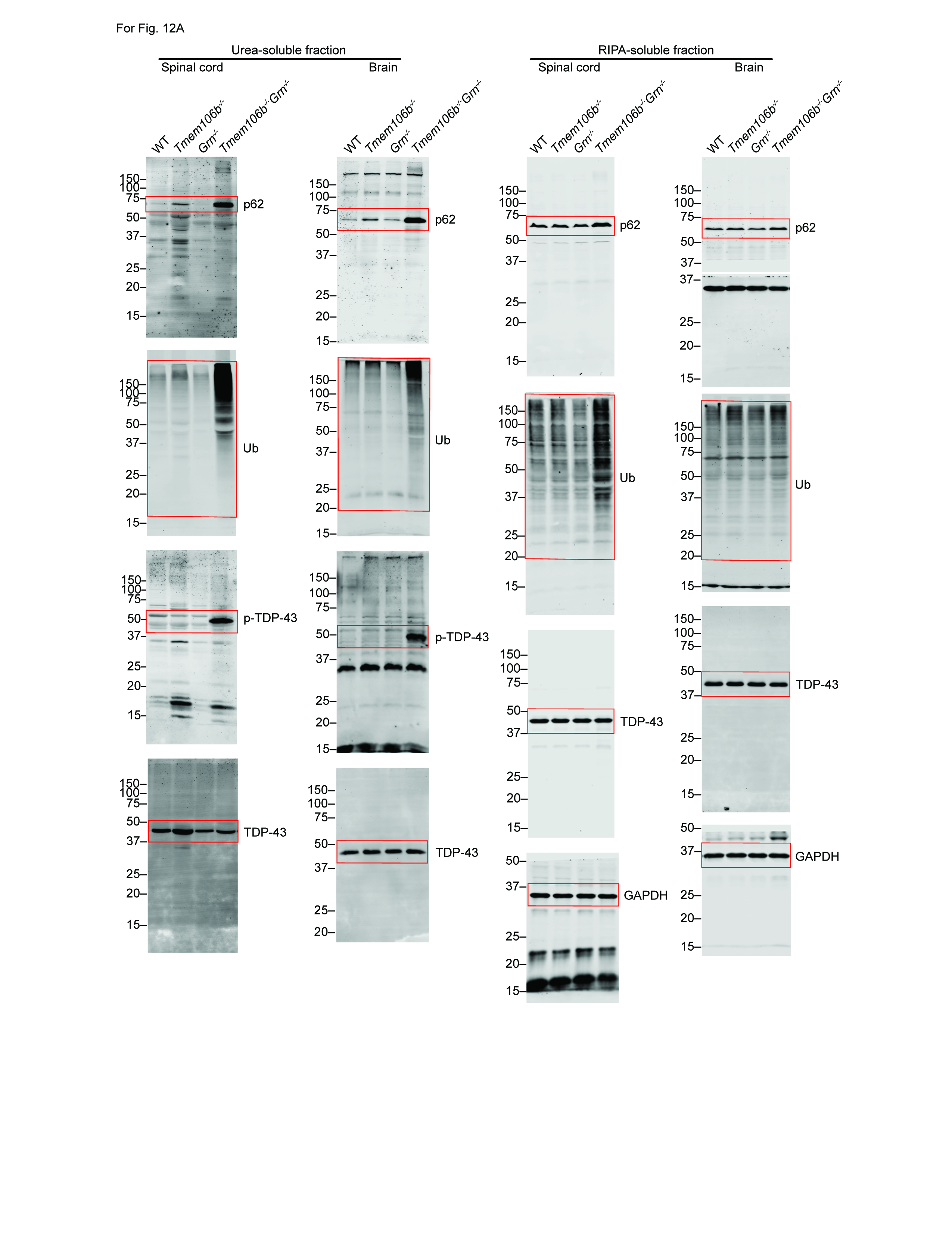

Supplement: Supplementary file 14 — Source Data for Figure 12 [file EMBR-21-e50219-s012.tif]
